# Enterohemorrhagic *Escherichia coli* effector EspF triggers oxidative DNA lesions in intestinal epithelial cells

**DOI:** 10.1128/iai.00001-24

**Published:** 2024-02-28

**Authors:** Yuting Fang, Muqing Fu, Xinyue Li, Bao Zhang, Chengsong Wan

**Affiliations:** 1 BSL-3 Laboratory, Guangdong Provincial Key Laboratory of Tropical Disease Research, School of Public Health, Southern Medical University, Guangzhou, Guangdong, China; 2 Guangzhou Institutes of Biomedicine and Health, Chinese Academy of Sciences, Guangzhou, China; University of California San Diego School of Medicine, La Jolla, California, USA

**Keywords:** DNA lesions, enterohemorrhagic *Escherichia coli*, EspF, 8-oxoguanine, RPA, DNA damage response

## Abstract

Attaching/effacing (A/E) pathogens induce DNA damage and colorectal cancer by injecting effector proteins into host cells via the type III secretion system (T3SS). EspF is one of the T3SS-dependent effector proteins exclusive to A/E pathogens, which include enterohemorrhagic *Escherichia coli*. The role of EspF in the induction of double-strand breaks (DSBs) and the phosphorylation of the repair protein SMC1 has been demonstrated previously. However, the process of damage accumulation and DSB formation has remained enigmatic, and the damage response is not well understood. Here, we first showed a compensatory increase in the mismatch repair proteins MutS homolog 2 (MSH2) and MSH6, as well as poly(ADP-ribose) polymerase 1, followed by a dramatic decrease, threatening cell survival in the presence of EspF. Flow cytometry revealed that EspF arrested the cell cycle at the G2/M phase to facilitate DNA repair. Subsequently, 8-oxoguanine (8-oxoG) lesions, a marker of oxidative damage, were assayed by ELISA and immunofluorescence, which revealed the accumulation of 8-oxoG from the cytosol to the nucleus. Furthermore, the status of single-stranded DNA (ssDNA) and DSBs was confirmed. We observed that EspF accelerated the course of DNA lesions, including 8-oxoG and unrepaired ssDNA, which were converted into DSBs; this was accompanied by the phosphorylation of replication protein A 32 in repair-defective cells. Collectively, these findings reveal that EspF triggers various types of oxidative DNA lesions with impairment of the DNA damage response and may result in genomic instability and cell death, offering novel insight into the tumorigenic potential of EspF.

**IMPORTANCE:**

Oxidative DNA lesions play causative roles in colitis-associated colon cancer. Accumulating evidence shows strong links between attaching/effacing (A/E) pathogens and colorectal cancer (CRC). EspF is one of many effector proteins exclusive to A/E pathogens with defined roles in the induction of oxidative stress, double-strand breaks (DSBs), and repair dysregulation. Here, we found that EspF promotes reactive oxygen species generation and 8-oxoguanine (8-oxoG) lesions when the repair system is activated, contributing to sustained cell survival. However, infected cells exposed to EspF presented 8-oxoG, which results in DSBs and ssDNA accumulation when the cell cycle is arrested at the G2/M phase and the repair system is defective or saturated by DNA lesions. In addition, we found that EspF could intensify the accumulation of nuclear DNA lesions through oxidative and replication stress. Overall, our work highlights the involvement of EspF in DNA lesions and DNA damage response, providing a novel avenue by which A/E pathogens may contribute to CRC.

## INTRODUCTION

Enterohemorrhagic *Escherichia coli* (EHEC) is a human foodborne pathogen that colonizes the colon and causes outbreaks of bloody diarrhea and hemolytic uremic syndrome worldwide (1). The formation of attaching and effacing (A/E) intestinal lesions is one of the major features of EHEC virulence and pathogenesis, which is dependent on the secretion of proteins by the type III secretion system (T3SS) (2). Oxidative stress and subsequent DNA lesions are considered as a starting point in the development of colitis-associated cancer (3). 8-Oxoguanine (8-oxoG) and phosphorylated histone H2A variant H2AX are commonly used as biomarkers for oxidative DNA damage and double-strand breaks (DSBs) in colorectal cancer (CRC) (4). Although the tumorigenic potential of EHEC has been previously discussed (5–7), there is limited evidence of the mechanisms underlying the formation of oxidative DNA lesions.

The DNA damage response (DDR) is a network of events in response to DNA damage, which includes DNA damage recognition, activation of checkpoints, cell cycle arrest, and eventually DNA repair or apoptosis (8, 9). It is controlled by a family of phosphoinositide 3-kinase-related kinases (PIKKs) that include ataxia-telangiectasia mutated (ATM) and ATM- and Rad3-related (ATR) (10). Structural maintenance of chromosomes protein 1 (SMC1) is a core component of the tetrameric complex cohesin and is normally localized to the nucleus, where it performs its biological functions (11, 12). Furthermore, it is phosphorylated by ATM and collaborates with the MRE11-Rad50-NBS1 (MRN) complex at DNA damage sites for DSB repair and activation of S phase arrest (13–15).

Mismatch repair (MMR) and base excision repair (BER) are two major repair mechanisms for oxidative DNA lesions (16–18). MMR is initiated through mismatch recognition by MutS homologs MSH2-MSH6 to prevent the accumulation of mutations (19). MSH2 was found to interact with ATR and regulate the phosphorylation of checkpoint kinase 1 (CHK1) and SMC1 (20, 21). Poly(ADP-ribose) polymerase 1 (PARP1) is the chief human PARP involved in DNA damage repair, including BER (17, 22, 23). It also acts as a first responder that detects DNA strand breaks and as a regulator that mediates the recruitment of the MRN complex and exonuclease 1 in MMR (23–25). Its catalytic activity, phosphorylation, and nuclear localization are regulated by ATM-checkpoint kinase 2 (CHK2), which is activated upon oxidative DNA damage (26).

Replication protein A 32 (RPA32) is one of the subunits of RPA. It is required for DNA replication and repair, and it binds to single-stranded DNA (ssDNA) that is generated during DNA resection (27, 28). Its phosphorylation by PIKKs is responsible for the recruitment of repair factors and the regulation of the cell cycle when DSBs and ssDNA occur (29, 30). In addition, its phosphorylation is also considered to be a hallmark of the replication stress response (29). The first and most prominent protein for which focus formation at the site of DSBs was described is H2AX, which is phosphorylated at Ser139 by PIKKs to generate γ-H2AX (31, 32). Moreover, RPA functions in the recognition of damaged DNA in the earliest stages of DDR while RPA phosphorylation and γ-H2AX are found at DSBs, which are a serious type of DNA damage and difficult to repair (32, 33).

Infection by A/E pathogens, including enteropathogenic *Escherichia coli* (EPEC) and EHEC, has been found to trigger the formation of DNA lesions and activate DDR in infected cells. UshA, a novel T3SS genotoxin in A/E pathogens, directly digests DNA substrates *in vitro*, triggers single-base substitutions and DSBs, and prompts colon tumorigenesis *in vitro* and *in vivo* (6). The T3SS-effector cycle inhibiting factor (Cif) blocks cell cycle progression at the G1/S and G2/M phase transition without DNA insult and DDR activation and eventually leads to cell death (34–36).

EspF, which belongs to a raft of T3SS-dependent effector proteins exclusive to A/E pathogens, has been shown to target and disrupt the nucleolus, impair mitochondrial function, and induce apoptosis (37, 38). EPEC EspF depletes MMR proteins and leads to the accumulation of mutations in microsatellite sequences, a condition known as microsatellite instability (MSI) (39, 40). In contrast to EPEC EspF, EHEC EspF was reported to increase intracellular reactive oxygen species (ROS) levels and promote apoptosis and inflammation through its N-terminal domain in previous work (41). We recently found that EHEC EspF led to multi-nucleation, hypertrophy, and phosphorylation of H2AX, core features of severe DNA damage. Moreover, the ATM downstream protein SMC1 was phosphorylated and shifted from the nucleus to the cytoplasm, inhibiting the activation of DDR (5). Interactions between EspF and mitotic arrest-deficient 2 like 2 (MAD2L2), the latter of which inhibits the Cdc20-related protein (Cdh1) and the anaphase-promoting complex, have been demonstrated, suggesting that EspF plays an essential role in cell cycle and mitotic control (7, 42–45). The characteristics and mechanisms of DNA lesions and the subsequent DDR during EHEC infection are not fully described, and the genotoxicity of EspF remains to be further demonstrated.

## RESULTS

### EspF impairs DNA damage recognition and cell cycle control, threatening cell survival

To investigate the effects of EHEC infection and EspF on DNA damage recognition proteins that initiate DNA repair, we established co-culture experiments using wild-type EHEC, an *espF*-deficient (Δ*espF*) mutant, and an *ΔespF* mutant complemented with plasmid-encoded *espF* (Δ*espF*/*pespF*) (46). We then determined the changes in the expression levels of DNA damage sensors in Caco-2 cells.

**Fig 1:**
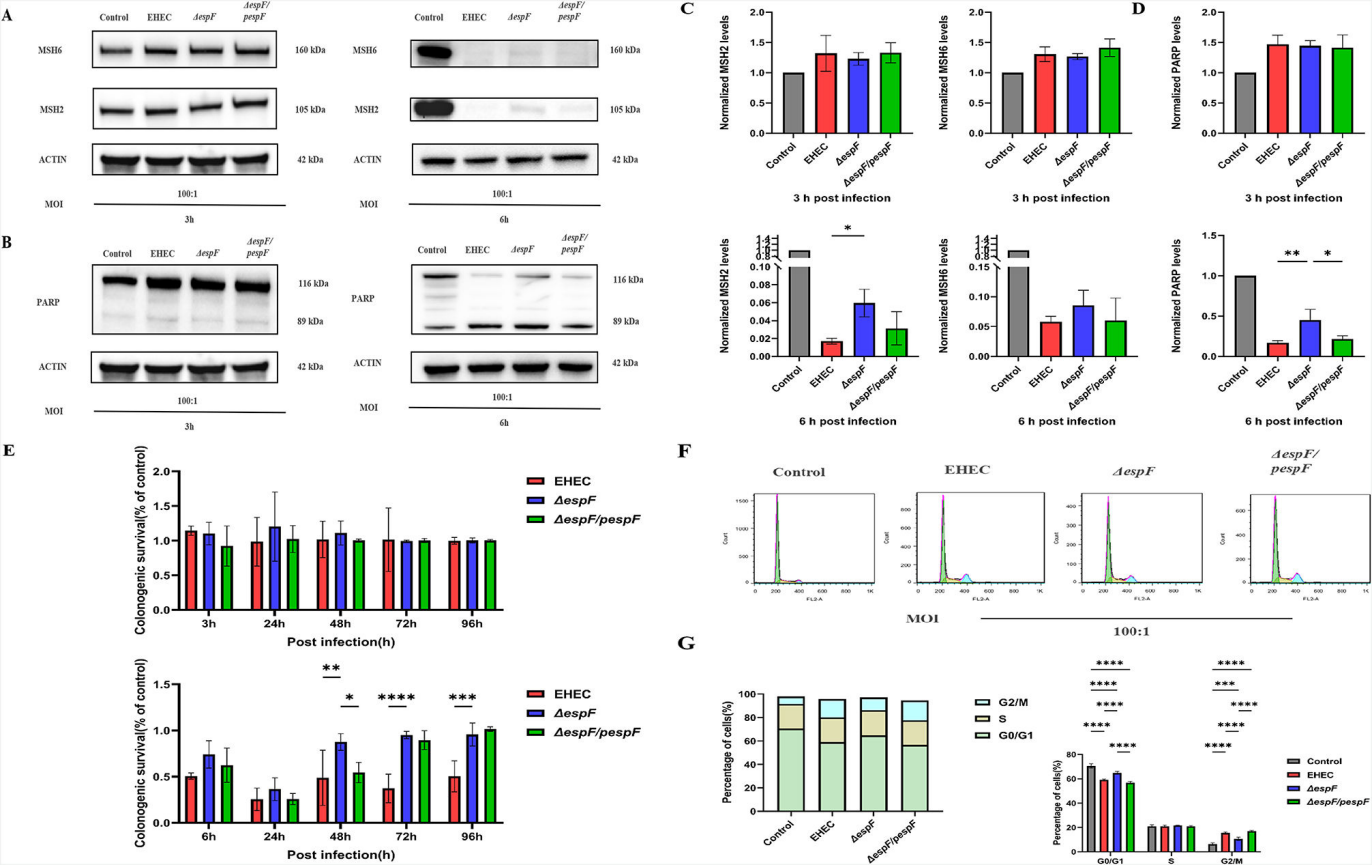
EspF impairs damage recognition and the cell cycle, threatening cell survival. (A–D) Expression levels of MSH2, MSH6(A), and PARP(B) 3 and 6 h after infection with EHEC EDL933w, Δ*espF,* and Δ*espF*/*pespF* as detected by western blot*.* Normalized MSH2, MSH6(C), and PARP(D) levels are shown. Values were normalized to β-actin expression levels. The samples collected at 3 hpi exhibited a shared expression level of β-actin. (**E**) Colony formation rate of cells treated with penicillin-streptomycin-gentamicin solution for up to 96 h after removal of any un-invaded bacteria. OD_595_ was measured and normalized to the control value (uninfected cells). (**F**) Distribution of the cell cycle after incubation for 9 h as assessed by PI staining and flow cytometry. (**G**) The percentage of cells in each phase. Data are expressed as mean ± SD from three independent experiments. *P < 0.05, **P < 0.01, ***P < 0.001, and ****P < 0.0001, one-way or two-way ANOVA and the chi-squared test.

Given that EPEC EspF causes depletion of MutS homolog (40), we speculate that EHEC EspF may be involved in MMR recognition. Immunoblots revealed that EHEC slightly elevates the protein expression of MSH2 and MSH6 at 3 h post-infection (hpi) in response to subsequent DNA lesions (Fig. 1A, left panel and Fig. 1C, upper panel). In contrast, EHEC causes a drastic reduction in the protein expression of MSH2 and MSH6 after 6 h of infection, which in turn impairs DNA repair. In Δ*espF*, the reduction in MSH2 and MSH6 levels was smaller, but the DNA repair capacity was not fully restored (Fig. 1A, right panel and Fig. 1C, lower panel). Changes in MSH6 expression may be a direct effect of infection or secondary to changes in MSH2 levels, as MSH6 stability is dependent on MSH2 expression.

Similarly, PARP1 was upregulated under stress conditions at 3 hpi and strongly downregulated at 6 hpi as expected (Fig. 1B and D). Our previous studies established that EHEC EspF induces apoptosis in infected HT-29 and Lovo cells (41, 47). Unexpectedly, infection with Δ*espF* resulted in the appearance of a stronger cleaved PARP band than that with EHEC and Δ*espF*/*pespF*. This may indicate that EspF permits cells to either adapt to the stress transiently, providing more opportunities for survival and replication for EHEC (48, 49), or promote apoptosis in response to irreversible damage.

The decision between cell survival and death following DNA damage rests primarily on factors that are involved in DNA damage recognition, DNA repair, and damage tolerance, as well as the activation of apoptosis (50). Clonogenicity is the ability of a single cell to grow into a colony that consists of at least 50 cells through cell proliferation (51, 52). Clonogenic experiments revealed that EHEC infection stimulated transient increases in clonogenicity and led to irreversible inhibition of colony formation due to its effector EspF after 48 h, supporting the idea that EspF poses a threat to cell survival due to an imbalance in DNA repair, which leads to observable damage at 6 hpi (Fig. 1E).

Considering that EspF may activate checkpoint and mitotic control through different mechanisms (7), we attempted to evaluate the cell cycle status through PI staining and flow cytometry. The proportion of cells in the G0/G1 phase showed a significant decrease in the EHEC group (59.22% ± 0.70%) and the Δ*espF*/*pespF* group (56.80% ± 1.19%), compared with the Δ*espF* group *(*64.81% ± 1.19%) and the control group (70.66% ± 1.84%). A significant increase in the proportion of cells in the G2/M phase was also detected in the EHEC group (15.52% ± 0.61%) and the Δ*espF*/*pespF* group (16.86% ± 0.77%) compared with the Δ*espF* group *(*10.79% ± 1.40%) and the control group (6.34% ± 1.09%) (Fig. 1F and G, *P* < 0.0001).

Taken together, these results indicated that EHEC induces cellular stress, accompanied by compromised repair systems. Under adaptive stress, EHEC induces a compensatory increase in the expression of response factors, which in turn enhances the recognition of DNA lesions and repair signaling that sustain cell survival in early-stage infection. However, EHEC EspF causes a dramatic depletion of these proteins and thus reduces the host’s capacity for DNA damage recognition, binding, and recruitment. This depletion drives G2/M cell cycle arrest by inducing oxidative stress, which in turn hampers the activation of the repair response, resulting in damage accumulation and, ultimately, cell death. Briefly, EspF might provide some level of interference with DNA damage recognition and the cell cycle, which are involved in cell survival, which was in agreement with our previous results.

### EspF promotes ROS generation and the formation of oxidative DNA lesions in repair-defective cells

**Fig 2:**
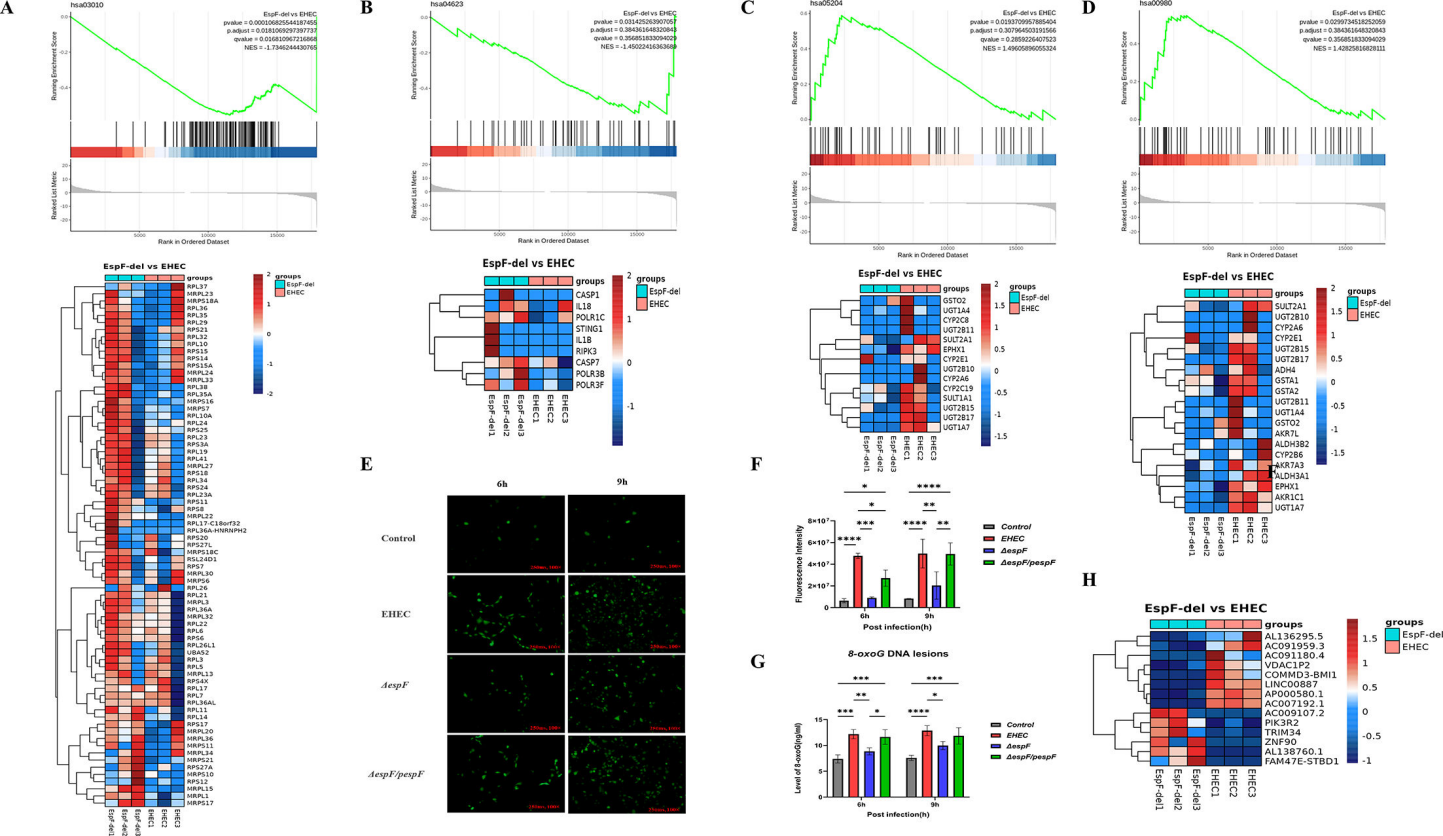
EspF promotes ROS generation and the formation of oxidative DNA lesions in repair-defective cells. Total RNA was extracted from cells infected with EHEC 933w (EHEC) and Δ*espF* (EspF-del) for 9 h and from uninfected cells in three biological replicates. RNA-seq was conducted to determine the differences in host response to wild-type EHEC and Δ*espF* at the transcriptional level. (A–D) Gene set enrichment analysis diagrams. Significant enrichment was determined using a *P*-value cutoff of 0.05 and a false discovery rate (FDR) of 0.25. (**E**) Representative fluorescence images of infected cells stained with DCFH-DA (marker for ROS, green) after 6 and 9 h of exposure to wild-type EHEC, Δ*espF,* and Δ*espF*/*pespF* (100×). (**F**) Mean fluorescence intensity of ROS. (**G**) The level of 8-oxoG DNA lesions was measured by ELISA. (**H**) Heatmap for differentially expressed genes (DEGs). Genes with log_2_ (fold change) ≥ 1 (upregulated, red) or log_2_ (fold change) ≤ 1 (downregulated, blue) and FDR-adjusted *P*-value < 0.05 were considered as DEGs. Data are expressed as mean ± SD from three independent experiments. *P < 0.05, **P < 0.01, ***P < 0.001, and ****P < 0.0001, one-way or two-way ANOVA.

To further investigate the variation in the expression levels of DNA repair genes and the association with cancer risk, we performed RNA-seq on control, EHEC, and *ΔespF* samples, followed by gene set enrichment analysis (GSEA). While there was no significant enrichment in multiple repair pathways, the ribosome pathway (hsa03010, adjusted *P*-value = 0.0316) stood out as the most significantly affected pathway (Fig. 2A). Considering the strong contribution of EHEC infection (uninfected group vs infected groups), other differences between the EHEC and *ΔespF* groups were small and we relaxed the criteria in subsequent analyses to identify more targets. Subsequently, the cytosolic DNA-sensing pathway (hsa04623), DNA adducts (hsa05204), and metabolism of xenobiotics by cytochrome P450 (hsa00980) were found (Fig. 2B through D). These results indicate that EspF may target the nucleus, impair ribosome biogenesis to inhibit translation and protein synthesis to deplete repair proteins, and promote cell cycle arrest or apoptosis activated by p53 (37, 53, 54), which, alongside the differential gene analysis results (Fig. 2H), supports its relevance to cancer.

Oxidative DNA lesions are widely considered as a potential risk factor for the development of CRC (55). We showed previously that EspF increases the production of ROS and induces the generation of γ-H2AX (5, 41), suggesting that signs of oxidative DNA lesions exist during EHEC infection. To explore this hypothesis, we determined the level of 8-oxoG, the most abundant DNA lesion after exposure to oxidative stress, which serves as an oxidative stress biomarker for CRC (4). Caco-2 cells were incubated with the indicated strains for 6 and 9 h, and a time-dependent increase in ROS levels dependent on EspF was observed. Meanwhile, large amounts of ROS were observed at 9 hpi, which could account for the high γ-H2AX observed at 9 h (Fig. 2E and F).

Then, DNA was extracted from Caco-2 cell lysates using the Tiangen DNA extraction kit to detect the levels of total 8-oxoG lesions by ELISA in infected cells. As expected, each group showed increased levels of 8-oxoG lesions, but there was only a modest increase in 8-oxoG lesions in the Δ*espF* group, which suggests EspF is involved in the formation of oxidative DNA lesions (Fig. 2G). Surprisingly, the level of 8-oxoG lesions induced by EHEC infection did not increase in a time-dependent manner, despite ROS accumulation, possibly due to the induction of other serious types of oxidative lesions or saturated 8-oxoG lesions. Another possible explanation could simply be alternations in the distribution of 8-oxoG lesions. These results show that EspF triggers the formation of oxidative DNA lesions as ROS accumulates.

### EspF intensifies the accumulation of oxidative DNA lesions in the nuclei

**Fig 3:**
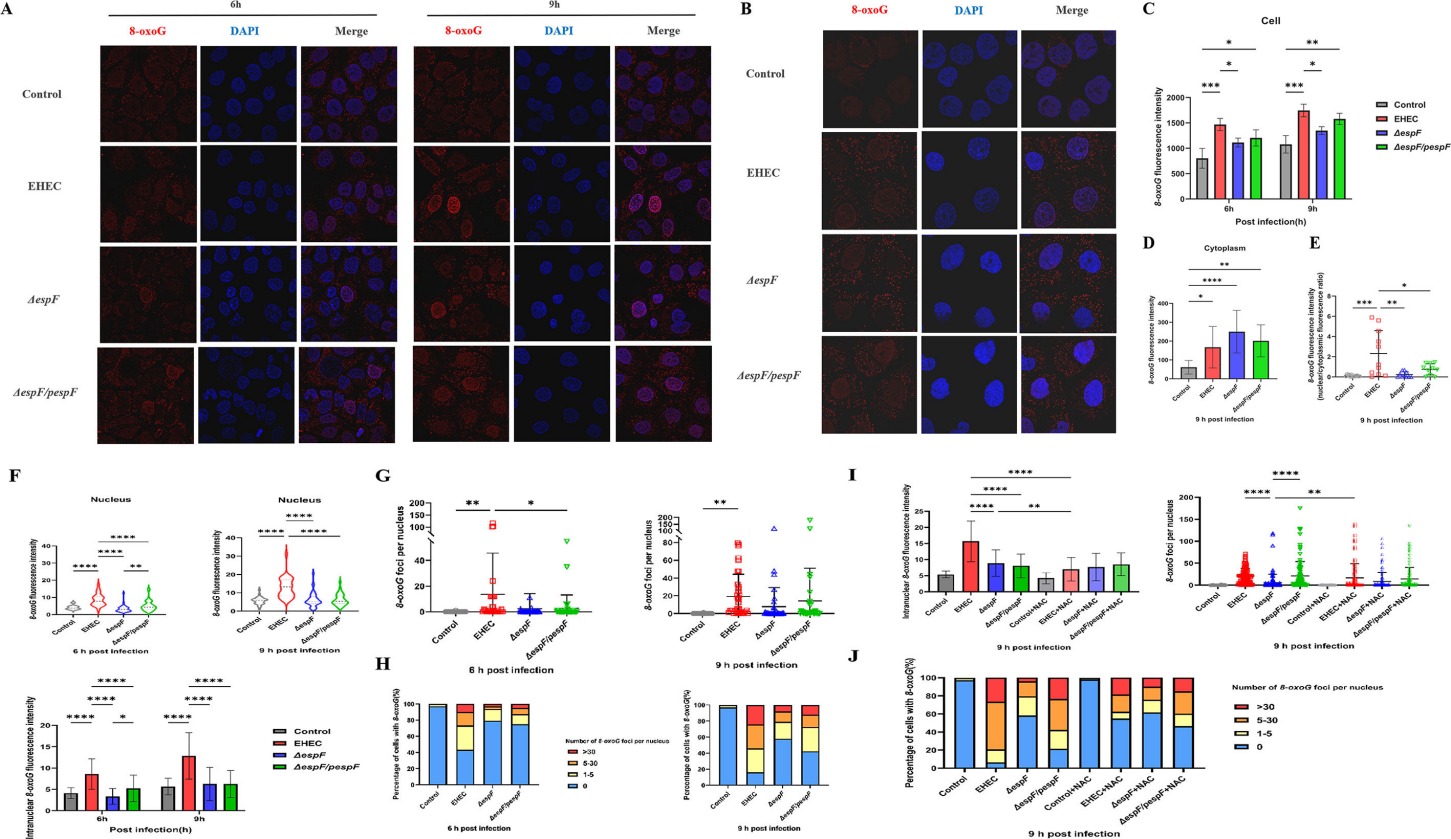
EspF intensifies the accumulation of oxidative DNA lesions in the nuclei. (**A**) Representative confocal microscopy images of cells infected with wild-type EHEC, Δ*espF*, and Δ*espF*/*pespF* for 6 and 9 h are shown (63× oil objective)*.* (**B**) Representative confocal image at 9 hpi (100× oil objective)*.* (C*–*F) The mean fluorescence intensity of 8-oxoG DNA lesions (red) in whole cells (**C**), which includes cytoplasm (D, 9 hpi) and nucleus (blue, **F**), and the ratio of mean fluorescence nucleocytoplasmic intensity (**E**) was calculated. (**G and H**) The foci of 8-oxoG DNA lesions per nucleus (**G**) and the percentage of positive cells (8-oxoG foci ≥ 5) and severely damaged cells (8-oxoG foci ≥ 30) (**H**) were quantified. Cells were treated with indicated strains for 9 h in the presence or absence of the ROS inhibitor NAC (10 mM). (**I and J**) The intranuclear 8-oxoG fluorescence intensity, the number of foci of 8-oxoG DNA lesions per nucleus (**I**), and the percentages of 8-oxoG*-*positive cells and severely damaged cells (**J**) are shown. For each sample, 30–40 cells or nuclei were randomly selected and counted. Data are expressed as mean ± SD from three independent experiments. *P < 0.05, **P < 0.01, ***P < 0.001, and ****P < 0.0001, one-way or two-way ANOVA and the chi-squared test.

Nuclear DNA damage is regarded as a major culprit in cancer (56). The distribution of 8-oxoG lesions (red) as assessed by immunofluorescence was found to be punctate in the nucleus (blue) and cytoplasm. The red fluorescence signal was weaker and present mostly in cytoplasmic and perinuclear regions after 6 h of infection. Notably, red fluorescence increased in intensity and was concentrated predominantly in foci, which were distributed throughout the nucleus at 9 h after infection (Fig. 3A). 8-oxoG lesions in the Δ*espF* group were in part localized at perinuclear regions and in part distributed within the nucleus; however, a predominantly homogenous nuclear distribution was observed in the EHEC and *ΔespF/pespF* groups (Fig. 3B). This suggests that EspF drives the formation of oxidative lesions in the nucleus rather than the cytoplasm.

Quantitative analysis revealed a reduction in the mean fluorescence intensity of 8-oxoG lesions in the Δ*espF* group*,* agreeing well with our ELISA data (Fig. 3C). The EHEC group exhibited a higher nuclear-to-cytoplasmic ratio than the Δ*espF* group (*P*  <  0.01); however, this does not appear to be associated with detectable damage of cytoplasmic DNA (Fig. 3D and E). In addition, cells infected with wild-type EHEC showed significant increases (vs Δ*espF*) in intranuclear 8-oxoG fluorescence intensity (*P* < 0.0001) and 8-oxoG foci-positive cells (*P* < 0.01), which was consistent with the trend of ROS we observed (Fig. 3F through H). To further evaluate the severity of damage, cells with more than 30 foci were classified as “severe damage.” Infection with wild-type EHEC and *ΔespF/pespF* caused a significant increase in the proportion of cells with severe damage, compared to infection with Δ*espF* (*P* < 0.01) (Fig. 3H). These observations confirmed that exposure to EHEC results in EspF-dependent accumulation of nuclear 8-oxoG lesions in a time-dependent manner.

**Fig 4:**
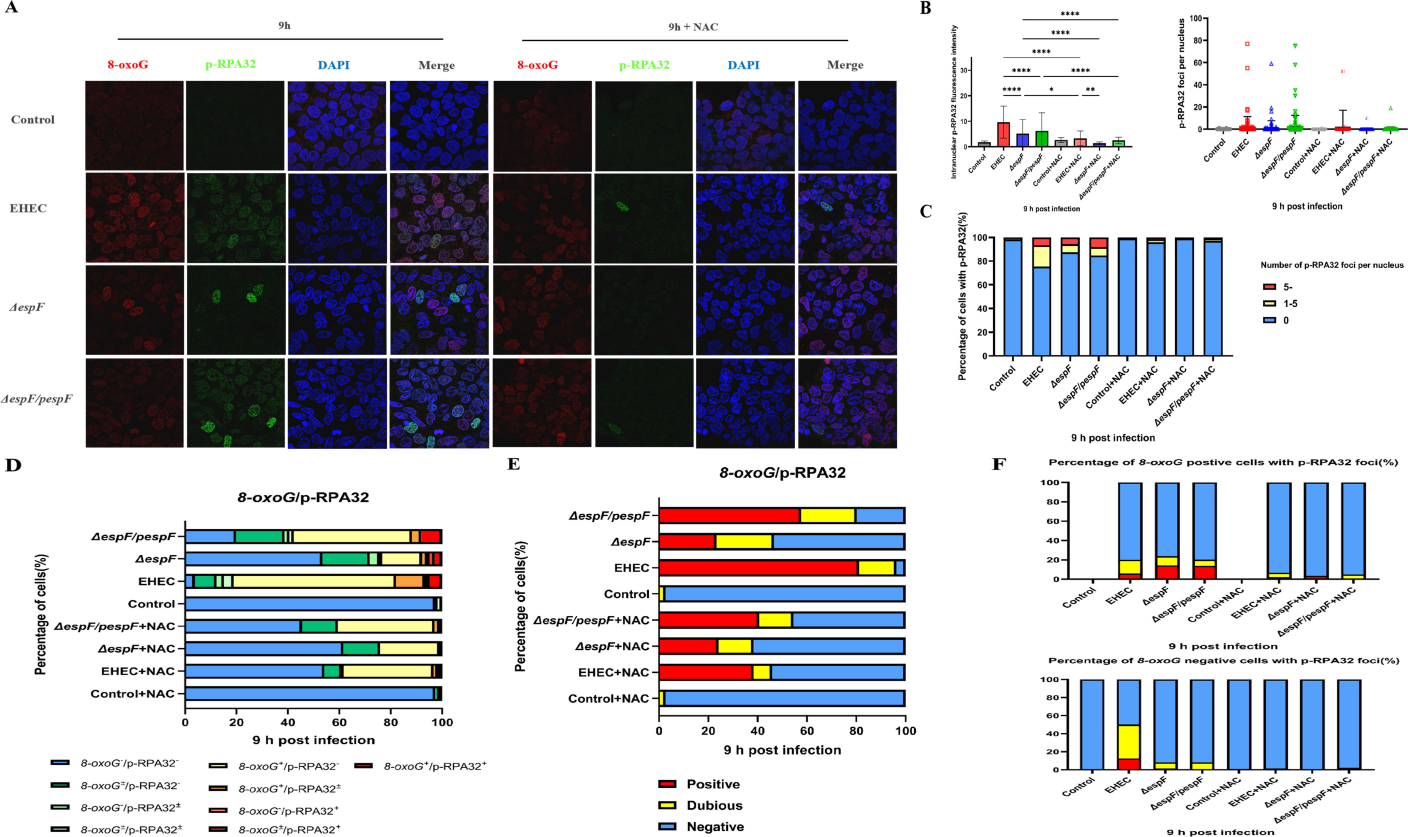
EspF induces a small increase in phosphorylation of RPA32. (**A**) Representative confocal microscopy images of cells stained for 8-oxoG DNA lesions (red) and p-RPA32 (green) after 9 h of infection with wild-type EHEC, Δ*espF*, and Δ*espF*/*pespF* with or without the ROS inhibitor NAC (10 mM) (63× oil objective). The intranuclear p-RPA32 fluorescence intensity, the number of foci of p-RPA32 per nucleus (**B**), and the percentages of positive cells (p-RPA32 foci ≥ 5) (**C**) were calculated. (**D and E**) The percentage of cells in each phase (**D**) or positive cells (**E**) with 8-oxoG/p-RPA32. (**F**) The percentage of cells with p-RPA32 foci in 8-oxoG-positive or -negative cells. For each sample, >100 cells or nuclei were randomly selected and counted. Data are expressed as mean ± SD from three independent experiments. *P < 0.05, **P < 0.01, and ****P < 0.0001, two-way ANOVA and the chi-squared test.

Given the observed oxidative stress and oxidative damage, we speculated that EspF may exert its effects on nuclear DNA by enhancing ROS production. Cells infected with indicated strains were preincubated with the antioxidant NAC (10 mM) for 1 h. Immunofluorescence experiments revealed that the levels of 8-oxoG lesions (red) in the nuclei of infected cells were markedly reduced by NAC treatment (Fig. 4A); however, there was no significant difference in intranuclear 8-oxoG fluorescence intensity and the number of 8-oxoG foci (Fig. 3I). Notably, loss of EspF resulted in a small proportion of positive and severely damaged cells compared to the wild-type EHEC and *ΔespF/pespF* groups (Fig. 3J). Collectively, EHEC could exert endothelial oxidative damage through ROS generation and the genotoxic effector protein EspF.

Overall, these results suggest that oxidative DNA lesions induced by EHEC accumulated in the nuclei, which were dependent primarily on ROS induction accompanied by impairment of oxidative damage repair. EspF was found to accelerate the accumulation of nuclear DNA lesions, which may directly lead to severe types of DNA damage and apoptosis or promote the production of ROS that indirectly exacerbate DNA damage.

### EspF induces a small increase in RPA32 phosphorylation

The protein kinases ATM and ATR are key DDR signaling components, which are recruited to and activated by DSBs and RPA-coated ssDNA, respectively (57). Considering that EHEC EspF interacts with the ATM substrate SMC1(17), we evaluated the phosphorylation status of RPA32 at Ser8 induced by ATM and DNA-dependent protein kinase (DNA-PK) at 9 hpi. The distribution of p-RPA32 (green) was found to be punctate in the nucleus (blue) (Fig. 4A).

In contrast to Δ*espF*, wild-type EHEC resulted in an increase in fluorescence intensity (*P* < 0.0001) but not in the number of foci of p-RPA32, a marker for replication stress, combined with ɣ-H2AX induction (5), demonstrating that EspF may induce replication stress and subsequent activation of DDR. When NAC was added, phosphorylation of RPA32 induced by EspF still occurred despite a significant decrease in fluorescence intensity (*P* < 0.01) (Fig. 4B and C).

The observation that 8-oxoG foci and p-RPA32 foci occurred within nuclei in infected cells further indicates that nuclear lesions were accompanied by replication stress and may give insight into the accumulation of damage. Of interest, the depletion of EspF decreased the proportions of positive and severely damaged cells, indicating EspF is essential for the progression of DNA lesions (Fig. 4D and E). Unexpectedly, loss of EspF did not result in lower levels of p-RPA32 in 8-oxoG*-*positive cells, while wild-type EHEC resulted in higher levels of p-RPA32 in 8-oxoG*-*negative cells (Fig. 4F). With regard to DSB formation and apoptosis activated by EspF, this would suggest that DNA was left severely damaged or perhaps resection was blocked, which contributed to the conversion of 8-oxoG into DSBs in damaged cells. Alternatively, EspF might cause a small amount of direct damage in undamaged cells.

### EspF accelerates the accumulation of oxidative DNA lesions and formation of DSBs

**Fig 5:**
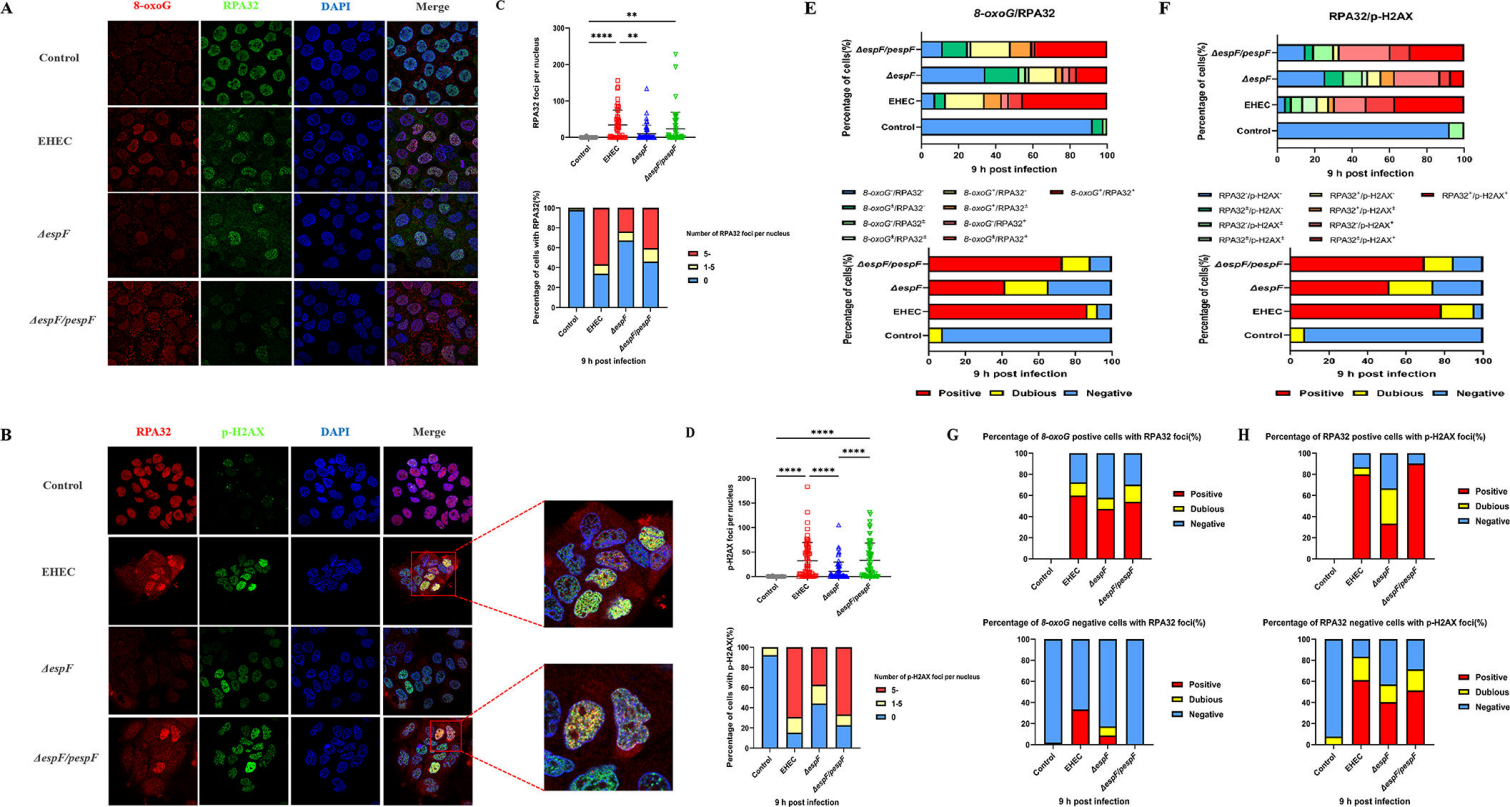
EspF accelerates the accumulation of oxidative DNA lesions and formation of DSBs. Cells were infected with wild-type EHEC, Δ*espF,* and Δ*espF*/*pespF* for 9 h. (**A and B**) Representative confocal microscopy images of indicated cells stained for 8-oxoG (red) and RPA32 (green) (**A**) or RPA32 (red) and p-H2AX (green) (**B**) (63× oil objective). (**C and D**) The number of foci of RPA (**C**) or p-H2AX (**D**) per nucleus and the percentage of positive cells (RPA32 foci ≥ 5 or p-H2AX foci ≥ 5) were calculated. (**E and F**) The percentage of cells in each phase or positive cells for 8-oxoG/RPA32 (**E**) or RPA32/p-H2AX (**F**). (**G and H**) The percentage of cells with RPA32 foci in 8-oxoG-positive or -negative cells (**G**) and with p-H2AX foci in RPA32-positive or -negative cells (**H**). For each sample, 60–70 cells or nuclei were randomly selected and counted for each sample. Data are expressed as mean ± SD from three independent experiments. **P  <  0.01 and ****P  <  0.0001, determined by one-way ANOVA and the chi-squared test.

To further understand the effect of oxidative DNA lesions in the nuclei, we co-stained treated cells for a marker of oxidized bases (8-oxoG), ssDNA (RPA32), and DSBs (p-H2AX) and measured the proportion of cells that contained indicated foci (Fig. 5A and B).

Immunofluorescence analysis of RPA32 recruitment in nuclear foci confirmed that cells infected with Δ*espF* displayed a marked decrease in the number of RPA32 foci and in the proportion of positive cells (*P* < 0.01) (Fig. 5C). This may indicate EspF causes ssDNA accumulation and exacerbates DSB formation by blocking resection repair (28). Consistent with previous findings, treatment with wild-type EHEC led to a significant increase in the number of p-H2AX foci and the proportion of positive cells compared to Δ*espF* (*P* < 0.001). Complementation of Δ*espF* with EspF restored its harmful effects (*P* < 0.01) (Fig. 5D). Interestingly, we observed colocalization of 8-oxoG with RPA32 foci and of RPA32 with p-H2AX foci in infected cells (Fig. 5A and B).

To account for the low levels of RPA32 phosphorylation and the cumulative effects of oxidative DNA lesions in damaged cells, the percentage of single-positive and double-positive cells was analyzed and the proportion of positive cells from damaged cells and undamaged cells was then compared. There were decreases in the percentage of positive cells (at least one of the damage types described above) and severely damaged cells (ssDNA or DSBs) in Δ*espF* compared with EHEC and *ΔespF/pespF*. As for the role of EspF in damaging DNA, it was evident that the magnitude of the changes at those transitions from buff to red in RPA32/p-H2AX damage foci was greater than that of 8-oxoG/RPA32 (Fig. 5E and F). Moreover, in the Δ*espF* group, 47.37% of 8-oxoG-positive cells had RPA32 foci and 33.33% of RPA32-positive cells had p-H2AX foci, whereas in the EHEC and *ΔespF/pespF* groups, 60% and 54.05% had RPA32 foci, and 80% and 90.48% had p-H2AX foci, respectively. Additionally, such an effect could be captured in undamaged cells, particularly via the generation of DSBs (Fig. 5G and H). Overall, these findings point to a central role for EspF in the accumulation of oxidative DNA lesions and suggest that EspF could give rise to limited amounts of DSBs in partial nuclear regions right at the outset as well as a gradual increase in the generation of DSBs resulting from oxidized bases and ssDNA in repair-defective cells.

## DISCUSSION

**Fig 6:**
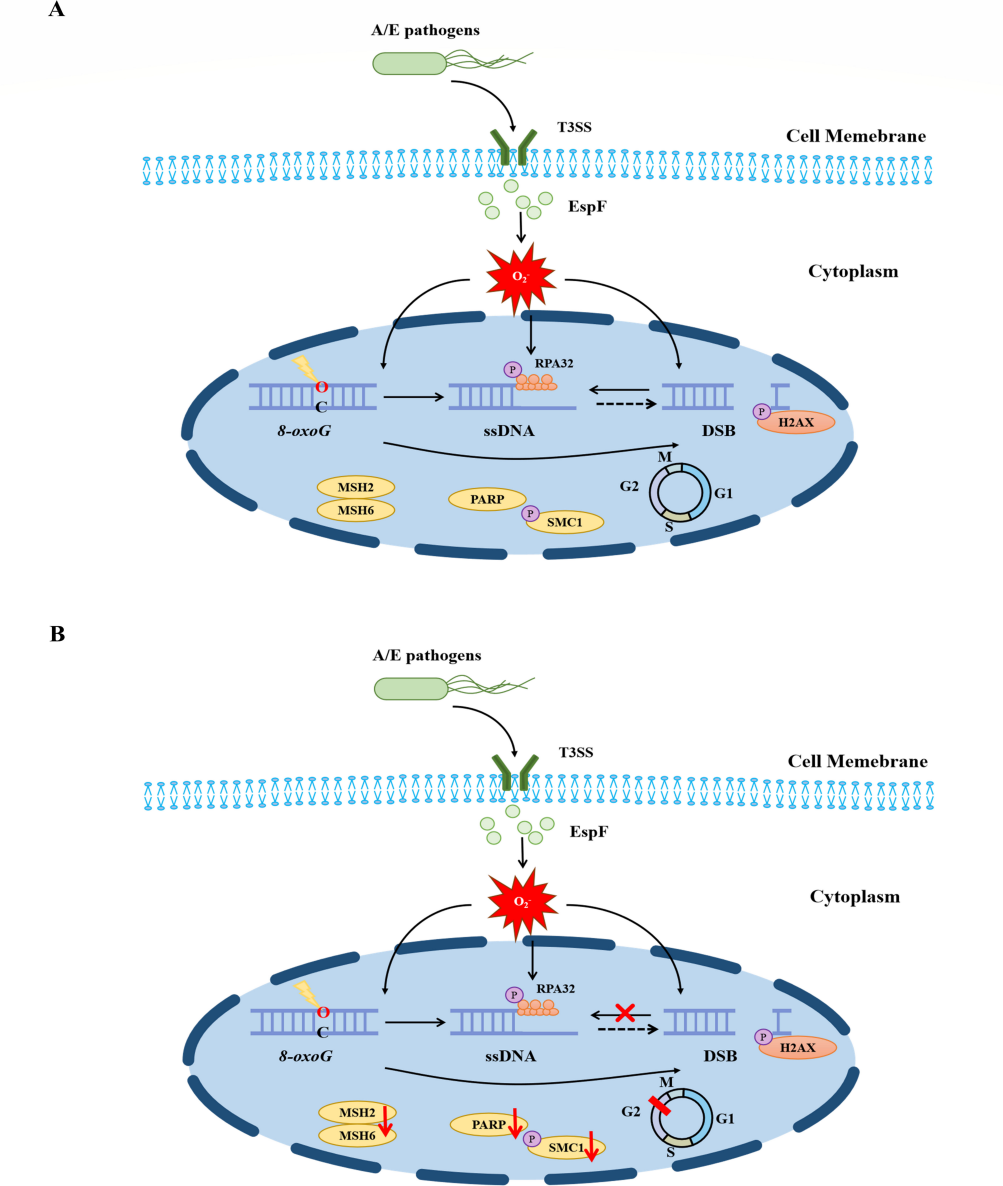
A model for the potential role of EspF in the induction of oxidative DNA lesions and activation of DDR. EspF is injected into host cells through the T3SS and it promotes the accumulation of substantial amounts of reactive oxygen species, resulting in 8-oxoG lesions and double-strand breaks. (**A**) The DNA damage response is activated in response to DNA lesions with the recruitment of repair proteins and the formation of RPA-coated single-stranded DNA (repair intermediates). (**B**) However, in addition to the accumulation of oxidative DNA lesions, unrepaired ssDNA persists and is converted into DSBs, while the repair system is saturated by DNA lesions or defective/inefficient despite G2/M cell cycle arrest to allow for DNA repair.

Numerous reports spanning nearly four decades of research have concluded that *E. coli*-produced genotoxins contribute to CRC in multiple forms of DNA damage or abnormal DDR (58). Although the tumorigenic potential of EspF in A/E pathogens was suggested by early evidence (5, 40, 59), our results show that EspF triggers a spectrum of DNA lesions comprising oxidized bases in response to oxidative stress and replication stress with impairment in DNA repair and the cell cycle in infected cells (Fig. 6). Tripartite motif 34, which sustains barrier integrity and attenuates colon inflammation and tumorigenesis (60), was downregulated in the presence of EspF consistent with the disruption of tight junctions (61–63).

Given the generation of ROS and the phosphorylation of H2AX elicited by EspF (5, 41), we attempted to detect 8-oxoG DNA lesions and demonstrated a time-dependent increase in ROS production and subsequent oxidative base modification in nucleoplasmic regions, likely as a result of disruption of the nucleolus by mitochondrial dysfunction (37). Furthermore, 8-oxoG DNA lesions first accumulated in the cytoplasm (or perhaps mitochondria) and then subsequently in the nucleus. This may not be surprising as mitochondrial DNA (mtDNA) is substantially more sensitive to oxidative damage than nuclear DNA, as mitochondria are deficient in efficient DNA repair mechanisms (64). In addition to accelerating the spread of damage, EspF intensifies the accumulation of nuclear 8-oxoG DNA lesions. Further work would be required to examine oxidative damage to GC-rich ribosomal DNA (65) or mtDNA.

Replication stress-driven ssDNA accumulation, followed by conversion into DSBs, is a new model of the carcinogenic effects of pathogenic *E. coli* strains (66, 67). A detailed analysis of the kinetics and type of DNA damage confirmed that induction of 8-oxoG in the early stages of infection with EspF is sufficient to induce ssDNA accumulation and conversion into DSBs in the late stages of infection. Interestingly, massive DSBs were formed in infected cells where excision was suppressed by phosphorylation of RPA32 (28) and thus irreparable ssDNA at replication forks could result in aberrant DNA structures and DSBs (68).

Small amounts of RPA32 phosphorylated at Ser8 by DNA-PK and ATM have been shown, arguing that EspF induces replication checkpoint arrest and may result in more persistent DSBs after release from replication stress (29). Given that crosstalk between these various kinases during RPA phosphorylation is complex and depends on the type of stress or damage and the cell cycle phase, Ser33, Ser4, and Thr21 phosphorylated by ATR and DNA-PK remain to be discovered in replication stress and strand breaks (30, 57). Moreover, NAC (10 mM) significantly attenuated 8-oxoG DNA lesions and RPA32 phosphorylation, which suggests the crucial role of ROS involved and does not exclude damage effects counteracted by high doses of NAC in part.

It is well established that DDR is a complex signal transduction pathway that has the ability to sense and remove DNA damage with the recruitment of repair factors, arrest cell cycle progression to allow for DNA repair and prevent the transmission of damaged DNA, and trigger apoptosis to eliminate heavily damaged or seriously deregulated cells (69, 70). As for MMR proteins, which are crucial for 8-oxoG and replication stress-associated DSBs, our data demonstrated a mild increase in response to infection and a dramatic depletion in response to persistent damage when the repair system is saturated, resulting in MSI in the presence of EspF (40, 71, 72). Similar phenomena also applied to PARP. Depletion of PARP was possibly due to auto-consumption by excessive activation in 8-oxoG lesions, indicating that EspF leads to PARP-dependent cell death triggered by the accumulated 8-oxoG in nuclear DNA (64). Nevertheless, whether EspF has any effect on the localization of PARP and its auto-modification remains to be seen (73, 74).

RNA-seq revealed that the ribosome pathway was inhibited in the wild-type EHEC group compared to the *ΔespF* group, suggesting that the inhibition of DNA repair by EspF may depend on the initiation of nucleolar stress featured by disruption of the nucleolus and impaired ribosome biogenesis (37, 53, 75, 76). Aside from acting as a signaling hub and as a sensor for oxidative and replication stress, the nucleolus is also considered to be an indispensable player in the regulation of the cell cycle and growth (75–77). Infected cells were found to arrest the cell cycle at the G2/M phase in the presence of EspF, preventing damaged cells from progressing into the M phase through RPA32 phosphorylation (30).

There are several limitations to our study. We assessed damage progression mostly based on damage repair foci detected by immunofluorescence; in the future, additional quantitative methods could be adopted. In addition, oxidative damage and its repair response are dynamic and complex. We attempted to select several time points to examine the effects in a staged manner. To maximize the observation of damage and minimize the impact of repair, the cell cycle, and apoptosis, we mostly used 9 h as the time point. Obviously, dynamic changes in oxidative damage and the recruitment of repair proteins have not been captured.

In conclusion, we found that infection by EHEC could trigger not only oxidative DNA lesions in the presence of EspF but also alterations in damage recognition, the cell cycle, and RPA phosphorylation. We also demonstrated that EspF triggers oxidative DNA lesions that are dominated by 8-oxoG and DSB lesions, dependent on oxidative and replication stress, in intestinal epithelial cells with defects in repair functions and G2/M arrest. Additionally, EspF exacerbates the accumulation of nuclear DNA lesions and broadens the range of DNA lesions from the cytoplasm to the nucleus. Thus, these findings delineate a novel role of EspF in inducing DNA lesions and tumorigenesis and provide evidence for links between EHEC and CRC.

## MATERIALS AND METHODS

### Cell lines and strains

EHEC strains O157:H7 EDL933w, *ΔespF*, and *ΔespF/pespF* were stored in our laboratory (41, 46). The bacterial strains were grown in Luria-Bertani medium in a shaker with kanamycin for *ΔespF* and chloramphenicol and L-arabinose for *ΔespF/pespF* at 37°C for 12 h (47).

Caco-2 cells were preserved in our laboratory and were grown in Dulbecco’s modified Eagle medium (DMEM) (Gibco) supplemented with 10% fetal bovine serum (FBS; ExCell Bio) in 5% CO_2_ at 37°C. For *in vitro* infections, cells were seeded in culture dishes or plates (NEST) and grown to a confluent monolayer.

### *In vitro* co-culture

Caco-2 cells were seeded 1 day prior to infection, and, just before infection, the supernatants were exchanged for fresh DMEM containing 2% FBS. Activated bacterial cultures were added to confluent cell monolayers at a multiplicity of infection of 100:1. After variable periods, cells were washed twice with PBS (Solarbio) and harvested by centrifugation.

### Western blot

Caco-2 cells were cultured in 10 cm diameter tissue culture dishes (approximately 1 × 10^7^ cells per dish) overnight and then infected with EHEC, Δ*espF*, and Δ*espF*/*pespF* for 3 and 6 h. Whole-cell protein extracts were prepared from cell pellets after lysis in radioimmunoprecipitation assay buffer (Beyotime) with protease inhibitor phenylmethanesulfonyl fluoride (PMSF) (Beyotime) and phosphorylase inhibitor cocktail (Solarbio). Equivalent amounts of protein from each sample were separated by SDS-PAGE in precast 8%–12% gradient FuturePAGE gels (ACE bio) and transferred to PVDF membranes (Millipore). Then, the membranes were blocked with 5% bovine serum albumin and incubated with rabbit anti-MSH2 (EPR21017-2) (diluted 1:1,000) (Abcam), rabbit anti-MSH6 (EPR3945) (diluted 1:1,000) (Abcam), rabbit anti-PARP (46D11) (diluted 1:1,000) (Cell Signaling Technology), and mouse anti-β-actin (diluted 1:10,000) (Proteintech) overnight at 4°C, followed by incubation with horseradish peroxidase-conjugated anti-rabbit or anti-mouse secondary antibody (Bioss) at room temperature. Finally, protein bands were visualized using hypersensitive chemiluminescence (ECL) reagent (Bioworld) and detected by a Tanon imaging system. Signal intensities were analyzed by ImageJ (v.1.52a). A single experiment was performed using the same batch of samples, normalized with a shared or corresponding internal control protein β-actin, to observe changes in the expression of target proteins.

### Colony formation assay

Caco-2 cells were seeded into 12-well plates at approximately 5 × 10^4^ cells/well 1 day prior to infection. The next day, cells were infected with EHEC, *ΔespF*, and *ΔespF/pespF* for 3 and 6 h. The non-adhered bacteria were aspirated, and the medium was replaced with a growth medium supplemented with Penicillin-Streptomycin-Gentamicin Solution (Solarbio). Cultures were continued for 96 h and media were replenished as necessary. At each time point, cell numbers were estimated based on the optical density at 595 nm (OD_595_) of solubilized crystal violet from 4% paraformaldehyde (Beyotime)-fixed cells.

### Flow cytometry

The cell cycle was analyzed by flow cytometry. The Caco-2 cells were grown in 6-well plates (approximately 1 × 10^6^ cells per well) overnight and then infected with EHEC, Δ*espF*, and Δ*espF*/*pespF* for 9 h. The cells were collected with pancreatin without EDTA, centrifuged, and washed with PBS. Then, the cells were collected with 0.25% pancreatic enzymes in the absence of EDTA and fixed in 70% ethanol overnight at −20°C. Next, Propidium Iodide/RNase Staining Buffer (Coolaber) was used for DNA staining. Cell cycle profiles were acquired with a FACSCalibur flow cytometer with CellQuest software (BD Bioscience).

### RNA isolation and RNA-seq

Caco-2 cells were infected with EHEC and *ΔespF* as described. Total RNA was extracted using AG RNAex Pro Reagent (Accurate Biotechnology) according to the manufacturer’s instructions. RNA quality evaluation, library construction, and sequencing were performed by APE ×Bio Technology LLC. Briefly, StringTie (v2.1.1) was used for gene and transcript quantification. Differential gene expression analysis and GSEA of the EHEC and *ΔespF* groups were performed with DESeq2 and ClusterProfiler (v4.2.2), respectively.

### Measurement of intracellular ROS and 8-oxoG lesions

Caco-2 cells were grown in 6-well plates (approximately 1 × 10^6^ cells per well) overnight and then infected with EHEC, Δ*espF*, and Δ*espF*/*pespF* for 6 and 9 h. The Reactive Oxygen Species Assay Kit (Beyotime) was used to determine ROS levels. After washing, the ROS probe DCFH-DA (diluted 1:1,000) was added to DMEM, and cells were incubated at 5% CO_2_ and 37°C. Stained cells were washed with PBS and observed immediately with a fluorescence microscope (TE2000-U, Nikon). ROS fluorescence was qualified and analyzed using ImageJ (v.1.52a). Cells were preincubated with the ROS inhibitor NAC (10 mM; Beyotime) 1 h prior to infection, followed by co-incubation with strains to assess the effect of endogenous and exogenous ROS. Genomic DNA was extracted from cell pellets using the TIANamp Genomic DNA kit (DP304) (Tiangen) according to the manufacturer’s instructions. The level of 8-oxoG lesions was assessed using a commercial competitive ELISA kit (INS-12324) (INSELISA) according to the manufacturer’s instructions.

### Immunofluorescence assay

Caco-2 cells were seeded in confocal dishes (35 mm; JETBIOFIL) at approximately 1 × 10^6^ cells per dish and co-cultured with EHEC, Δ*espF*, and Δ*espF*/*pespF* for 6 and 9 h. Cells were gently washed with PBS twice and fixed in 4% paraformaldehyde (Beyotime) for 15 min at room temperature. Then, the fixed cells were washed with PBS, supplemented with 0.1% Triton X-100 (Sigma-Aldrich), and blocked with 10% goat serum (Boster). Subsequently, each dish was incubated with anti-8-oxoG (483.15) (diluted 1:250) (Santa Cruz), anti-p-RPA32/RPA2 (Ser8) (E5A2F) (diluted 1:250) (CST), anti-RPA32/RPA2 (E8X5P) (diluted 1:800) (CST), and anti-p-histone H2A.X (Ser139) (D7T2V) (diluted 1:200) (CST) overnight at 4°C. Next, the cells were incubated with goat anti-mouse IgG Alexa Fluor 594, goat anti-mouse IgG Alexa Fluor 488, goat anti-rabbit IgG Alexa Fluor 594, or goat anti-rabbit IgG Alexa Fluor 488 (diluted 1:250) (Proteintech) in the dark for 30 min at room temperature. The samples were washed with PBS and nuclei were stained using DAPI (Beyotime). Finally, confocal images were taken with a 63× oil immersion objective with an LSM880 confocal microscope (Zeiss). The mean intensity of the fluorescence signal and the number of nuclear foci per cell were quantified using ImageJ (v.1.52a).

For each sample, 30–100 cells or nuclei were randomly selected and counted. Damage focus-positive cells were defined as cells with 8-oxoG foci ≥ 5, p-RPA32 foci ≥ 5, RPA32 foci ≥ 5, and γ-H2AX foci ≥ 5. The foci of DNA lesions per nucleus were scored as (0) no lesions/negative (4); 1–5 foci (dubious); or (29) ≥5 foci (positive).

### Statistical analysis

All experiments were performed at least thrice. Statistical analyses were carried out and graphs were generated using GraphPad Prism 9.0. Data are expressed as mean ± SD. Multiple comparisons were made using one-way or two-way ANOVA, and contingency testing was done with the chi-squared test. The level of significance was *P* < 0.05.

## Data Availability

The majority of the data supporting our conclusions are included in the article, and further inquiries can be directed to the corresponding authors. The RNA seq data generated herein are available from the Gene Expression Omnibus (GEO accession number GSE255129).
